# Functional ovarian cysts in a neonate with classical 21-hydroxylase deficiency: case report and review of the literature

**DOI:** 10.1186/1687-9856-2013-S1-P135

**Published:** 2013-10-03

**Authors:** Hideyuki Iwayama, Haruo Mizuno, Yutaro Hayashi, Tomonobu Hasegawa, Takeshi Usui

**Affiliations:** 1 Department of Pediatrics and Neonatology, Japan; 2 Department of Nephro-Urology, Nagoya City University Graduate School of Medical Sciences, Japan; 3 Department of Pediatrics, Keio University School of Medicine, Japan; 4 Department of Endocrinology and Metabolism, National Hospital Organization Kyoto Medical Center, Japan

## 

Ovarian cysts (OCs) have been described in patients with congenital adrenal hyperplasia (CAH), however, neonatal OCs are rare in CAH. We report a unique neonate with 21-hydroxylase deficiency (21OHD), having functional OCs which developed after the start of treatment for CAH. The patient was referred for clitoromegaly at age of 5 days. Because of elevated ACTH, 277 pg/ml; 17-alpha-hydroxyprogesterone (17OHP), 184.4 ng/ml; plasma rennin activity (PRA), 39 ng/ml/hr; testosterone, 4.58 ng/ml, classical salt-wasting 21OHD was diagnosed. Estradiol (E2) and gonadotropins were normal (E2 <10 pg/ml, LH 0.1 IU/l, FSH 0.2 IU/l). PCR direct sequencing revealed homozygosity for IVS2-13A/C>G in CYP21A2. MRI showed right adrenal bleeding, left adrenal hyperplasia, and no OCs. Hydrocortisone, fludrocortisone and sodium chloride were initiated. At age of 57 days, re-examination of MRI detected left OC (41 x 49 mm) and reduction of adrenal bleeding. E2 was remarkably elevated (939 pg/ml) and gonadotropins were not considerably increased (LH 0.5 IU/l, FSH 1.1 IU/l). ACTH, 17-OHP, PRA and androgens were not elevated. At age of 72 days, genital bleeding was seen for 5 days, followed by 5 times of genital bleeding per month. At age of 85 days, MRI showed development of right OC (54 x 36 mm) and reduction of the left one. Blood examination revealed E2, 99 pg/ml; LH, 0.3 IU/l; FSH 4.8 IU/l. At age of 10 months, OCs spontaneously diminished and E2 became under sensitivity. We determined that she had functional OCs. Treatment for 21OHD may give rise to them. To our best knowledge, including our case, 7 cases of neonatal OCs in CAH have been reported in the literature. Of these cases, 4 were diagnosed to have functional OCs after the initiation of treatment for CAH. In the 4 patients, all had genital bleeding and 3 had spontaneous regression of OCs. The remaining 3 cases had congenital OCs without genital bleeding and received cystectomy. Therefore, the mechanism of neonatal functional OCs in CAH may be different from that of congenital OCs and spontaneous regression could be expected without surgery.

